# The effect of sibutramine prescribing in routine clinical practice on cardiovascular outcomes: a cohort study in the United Kingdom

**DOI:** 10.1038/ijo.2015.86

**Published:** 2015-06-09

**Authors:** J F Hayes, K Bhaskaran, R Batterham, L Smeeth, I Douglas

**Affiliations:** 1 Division of Psychiatry, University College London, London, UK; 2 Department of Non-Communicable Disease Epidemiology, London School of Hygiene and Tropical Medicine, London, UK; 3 Department of Medicine, University College London, London, UK; 4 Department of Non-Communicable Disease Epidemiology, London School of Hygiene and Tropical Medicine, London, UK

## Abstract

**Background/Objectives:**

The marketing authorization for the weight loss drug sibutramine was suspended in 2010 following a major trial that showed increased rates of non-fatal myocardial infarction and cerebrovascular events in patients with pre-existing cardiovascular disease. In routine clinical practice, sibutramine was already contraindicated in patients with cardiovascular disease and so the relevance of these influential clinical trial findings to the ‘real World' population of patients receiving or eligible for the drug is questionable. We assessed rates of myocardial infarction and cerebrovascular events in a cohort of patients prescribed sibutramine or orlistat in the United Kingdom.

**Subjects/Methods:**

A cohort of patients prescribed weight loss medication was identified within the Clinical Practice Research Datalink. Rates of myocardial infarction or cerebrovascular event, and all-cause mortality were compared between patients prescribed sibutramine and similar patients prescribed orlistat, using both a multivariable Cox proportional hazard model, and propensity score-adjusted model. Possible effect modification by pre-existing cardiovascular disease and cardiovascular risk factors was assessed.

**Results:**

Patients prescribed sibutramine (*N*=23 927) appeared to have an elevated rate of myocardial infarction or cerebrovascular events compared with those taking orlistat (*N*=77 047; hazard ratio 1.69, 95% confidence interval 1.12–2.56). However, subgroup analysis showed the elevated rate was larger in those with pre-existing cardiovascular disease (hazard ratio 4.37, 95% confidence interval 2.21–8.64), compared with those with no cardiovascular disease (hazard ratio 1.52, 95% confidence interval 0.92–2.48, *P*-interaction=0.0076). All-cause mortality was not increased in those prescribed sibutramine (hazard ratio 0.67, 95% confidence interval 0.34–1.32).

**Conclusions:**

Sibutramine was associated with increased rates of acute cardiovascular events in people with pre-existing cardiovascular disease, but there was a low absolute risk in those without. Sibutramine's marketing authorization may have, therefore, been inappropriately withdrawn for people without cardiovascular disease.

## Introduction

Before 2010 sibutramine and orlistat were the two European Union approved pharmaceutical options for weight loss treatment for individuals with a body mass index (BMI) over 27 kg m^−2^.^1^ In January 2010, sibutramine was suspended following a review by the European Medicines Agency, who found an ‘increased risk of non-fatal myocardial infarction (MI) and stroke, which outweighed the possible benefits of medication through weight loss'.^2^ The United States Food and Drugs Administration followed suit later that year. Although concerns about sibutramine's safety had been raised before, the risk was clarified by the Sibutramine Cardiovascular Outcomes Trial (SCOUT),^3^ a randomized, placebo-controlled trial of over 10 000 patients with increased risk of cardiovascular events (that is, individuals with pre-existing cardiovascular disease or with type 2 diabetes mellitus (T2D) and cardiovascular risk factors). The aim of SCOUT was to clarify the cardiovascular and cerebrovascular side effect profile of sibutramine. The primary outcome was a composite of non-fatal MI, non-fatal cerebrovascular event (CVE), resuscitation after cardiac arrest, and cardiovascular death.^3^ The rate was increased by 16% in the sibutramine group compared with placebo (hazard ratio (HR) 1.16, 95% confidence interval (CI) 1.03–1.31, *P*=0.02), with overall incidences of 11.4% and 10.0%, in the two groups, respectively. This increased rate was made up of non-fatal events, rather than cardiovascular deaths.^3^

A number of limitations of this trial, especially related to its generalizability, have been identified: the increased risk was only shown in the groups with pre-existing cardiovascular disease, this was already a contraindication in prescribing guidance;^1^ all patients were aged over 55 years; the trial showed only a small increase in rates, especially when compared with other factors such as smoking; in certain groups (young women for example), a statistically significant increase in rate ratio may be unimportant in absolute terms;^4^ individuals who achieved modest weight loss associated with the 6-week sibutramine run-in period in SCOUT had reduced 5-year MI and CVE rates;^5^ prescribing of sibutramine in the trial did not reflect ‘real World' use, it continued for five times the licensed duration of treatment and the dose was not modified if the patient failed to lose weight;^4, 6, 7, 8^ there is little evidence that the alternative weight loss medication (orlistat) reduces cardiovascular risk,^4^ so a head-to-head comparison of these medications may be more informative.

Given the increasing levels of obesity worldwide, a better understanding of the ‘real World' effects of weight loss medications is needed. Between first licensing and suspension, a large number of patients in the United Kingdom were prescribed sibutramine, and use of data routinely gathered on these patients could address some of the limitations of SCOUT, while putting no further patients at risk. Thus, in this study, we aimed to assess the comparative risk of sibutramine and orlistat in ‘real World' use. Analysis of this cohort has shown that there is unlikely to be a clinically meaningful difference between these drugs in terms of weight loss; patients prescribed orlistat lost an average of 0.94 kg per month (95% CI 0.93–0.95) and patients prescribed sibutramine lost 1.28 kg per month (95% CI 1.26–1.30) over the first 4 months, but orlistat was slightly superior at sustaining weight loss at 3 years.^9^

Objectives of the study were to determine the rate of incident MI or CVE (both fatal and non-fatal) in patients prescribed sibutramine or orlistat, the rate of MI or CVE in high-risk patients (that is, those with pre-existing cardiovascular disease or T2D plus another cardiac risk factor) and the rate of all-cause mortality.

## Subjects and methods

### Study design

A cohort study using prospectively collected routine primary care data from the UK Clinical Practice Research Datalink (CPRD).

### Data source

CPRD contains anonymized healthcare records for ~14 million patients registered at over 660 general practice (GP) surgeries in the United Kingdom.^10^ This represents nearly 10% of the UK population.^11^ The database began in 1987 and continuously records information relating to each consultation. It contains sociodemographic data, prescribed medication records and Read Codes (searchable clinical terms) relating to diagnoses (made in primary and secondary care), signs and symptoms, and procedures and clinical investigations.^12, 13, 14^ CPRD has been shown to be largely representative of the United Kingdom in terms of GP surgery size and geographical distribution. Individuals registered in the database are representative in terms of age and sex.^15^

### Study participants

The cohort was drawn from CPRD starting from the date which the GP data was defined as being of suitable research quality^16^ and ending at the start of June 2013. Patients were included if they: were aged over 18 years old, were prescribed either sibutramine or orlistat, were recorded as having BMI ⩾27 kg m^−2^ (in line with NICE guidance for prescribing weight loss medication), had at least 12 months registration before their first prescription (to identify incident rather than prevalent users) and the data quality in their record had met minimum agreed standards for use in research.^16^ A subgroup analysis was completed on patients that before their first prescription of weight loss medication had a Read code consistent with a history of cardiovascular disease (not including acute MI or CVE) such as coronary artery disease, transient ischemic attack, or peripheral arterial occlusive disease, or T2D with at least one other cardiovascular risk factor (hypertension, dyslipidaemia, current smoking or diabetic nephropathy).

### Exposures, outcomes and covariates

Patients were defined as exposed during the time they received their first continuous period of sibutramine prescribing; from the date of the first prescription to the date of the last prescription, plus the prescription length (expected end of treatment date), plus 60 days (to account for possible delayed side effects of treatment). The comparison group were patients prescribed orlistat, with the exposure period defined in the same manner. Patients were censored at the earliest of: event date, death date, bariatric surgery date or end of first constant prescribing period.

The decision to define patients as exposed 60 days following final dose of sibutramine was made because the postulated mechanism for causing MI or CVE is via acute changes in heart rate, blood pressure and QT interval.^17^ This approach should, therefore, produce an overestimate of the risk of sibutramine.

The primary outcome of interest was time to first MI or CVE (MI/CVE). If a patient had multiple codes representing acute MI or CVE on multiple dates in their record the event was recorded as occurring on the date of first entry of that code. A secondary outcome was all-cause mortality; death was ascertained from patients' medical records and the date of death defined as the earliest of any records indicating that death had occurred.

Other covariates were examined as possible confounders: age, sex, BMI, year of index prescribing of weight loss drug, smoking status, alcohol consumption, comorbidities (history of coronary heart disease, cerebrovascular disease (CVD), peripheral vascular disease, any other atheroma, T2D and hypertension) and co-prescribing (oral anti-glycaemic medication, insulin and statin).

BMI, comorbidities and co-prescribing information was taken from the most proximate entry in the notes before the start of weight loss medication prescribing. Smoking and alcohol history were from the most proximate entry either before or after the start of prescribing. Patients with missing information on smoking and/or alcohol consumption were compared with those with complete data. A complete case analysis was performed as we found no evidence that missingness was associated with the outcome.^18^

### Sample size

Assuming a baseline 7% annual event rate,^3^ a two-sided type I error rate of 0.05, and power set at 90%, to detect a 10% difference in HR between orlistat and sibutramine would require a sample size of 19 000 patients prescribed sibutramine and 58 000 patients prescribed orlistat (with a 1:3 exposed to unexposed ratio). A preliminary analysis revealed 23 927 (23.70%) were exposed to sibutramine and 77 047 to orlistat. Given these numbers and the predicted number of events, there should be ample power to explore the main hypothesis.

### Statistical analysis

A multivariable Cox model was constructed to assess the association between sibutramine and both outcomes (MI/CVE, all-cause mortality) controlled for all other covariates considered, and adjusted for clustering within GP surgeries. Wald tests were used to calculate *P*-values for multivariable models. These results were compared with a propensity score (PS) analysis. A PS is a measure of the probability that a patient will receive a particular treatment and is calculated from the observed risk factors for the outcome and for receiving the treatment.^19^ This approach is, therefore, one possible solution to the problem of confounding by indication in observational studies. Covariates were included in the PS if they did not introduce multicollinearity in the logistic regression model predicting treatment allocation. The PS was included in a Cox regression model as a continuous variable.

### Subgroup analysis

Two subgroups were defined *a priori* based on the analysis of SCOUT; patients with pre-existing cardiovascular disease (7761 individuals) and patients with T2D plus at least one other cardiovascular risk factor (that is, hypertension, statin use and current smoking) (15 455 individuals). Effect modification because of possible interaction between sibutramine prescribing and existing cardiovascular disease or T2D plus other cardiac risk factors was examined using the PS-adjusted model.

If there were differences between subgroups these were presented as number needed to harm. Number needed to harm was calculated from the estimated survivor function generated from the PS-adjusted Cox regression model.^20^

### Model checking and sensitivity analysis

Assumption of proportional hazards was checked by producing Aalen plots and testing whether HRs varied over different intervals of time. A number of sensitivity analyses were completed (1) PS 1:1 matching, rather than using the score directly in the regression model; (2) censoring all patients prescribed orlistat at the date of sibutramine withdrawal (2010); and (3) assuming that the possible delayed effects of sibutramine lasted 15 days after the final dose, rather than 60 days. All analysis was carried out using Stata version 13.^21^

### Ethical approval

Ethical approval for this study was obtained from the London School of Hygiene and Tropical Medicine Ethics Committee and scientific approval was gained from the Independent Scientific Advisory Committee for Medicines and Healthcare products Regulatory Agency.

## Results

### Patient characteristics

In total, 100 974 individuals were included in the analysis, 23 927 (23.70%) were exposed to sibutramine and 77 047 to orlistat. Median age was 46.09 years (interquartile range (IQR) 36.42–56.66) median BMI was 36.36 kg m^−2^ (IQR 32.83–40.91). Patients prescribed sibutramine were more likely to be female (82 vs 76%), to take the medication for less time (0.39 vs 0.47 years) and not have cardiovascular risk factors (Table 1).

### Rate of incident MI or CVE

There were 254 incident MI/CVEs in the cohort; 34 in 0.126 per 100 000 person-years at risk (10^5^ PYAR) in patients exposed to sibutramine and 220 in 1.092 per 10^5^ PYAR in the orlistat group. In those exposed to sibutramine, the rate of MI/CVE was 269.39.12 per 10^5^ PYAR (95% CI 194.39–384.68). This was not different from those unexposed (crude HR 1.19, 95% CI 0.80–1.76, *P*=0.39).

**Figure 1:**
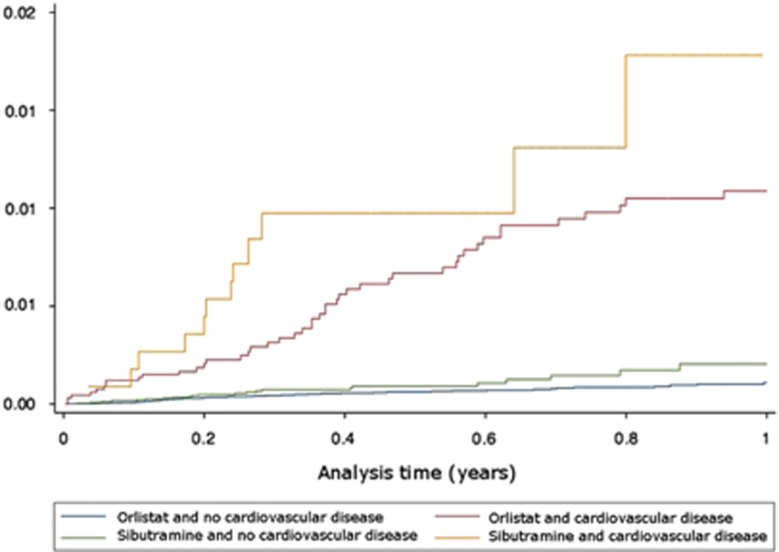
Interaction between sibutramine and history of cardiovascular disease.

The fully adjusted model accounting for age, sex, BMI, smoking status, alcohol use, cardiovascular disease, T2D and hypertension, as well as clustering by GP surgery gave a HR of 1.65 (95% CI 1.08–2.51, *P*=0.018). The PSs were similar for both sibutramine (median 0.26, IQR 0.22–0.28) and orlistat groups (median 0.24, IQR 0.20–0.28; Appendix 1). The model adjusting for PS showed a similar HR to the fully adjusted model (HR 1.69, 95% CI 1.13–2.54, *P*=0.011; Table 2). There was evidence that the effect was stronger in people with concurrent cardiovascular disease (HR 4.37, 95% CI 2.2–8.64) than those without (HR 1.52, 95% CI 0.92–2.48, *P*-interaction=0.0076). There was no evidence that the effect differed by T2D plus one other cardiac risk factor (that is, hypertension, statin use and current smoking) status (Table 2, Figure 1).

In patients with cardiovascular disease prescribed sibutramine, the number needed to harm (NNH) at 4 months (close to the median exposure time for sibutramine) was 129 (95% CI 57–360) and at 1 year was 28 (95% CI 12–77; Table 3).

PS matching on a 1:1 nearest neighbor basis (0.01 caliper), dropped 53 120 patient-treatment periods that were not matched, and only included 80 events. This method gave a HR of 1.63 (95% CI 0.91–2.92, *P*=0.10). Although the point estimate was similar, this method markedly reduced power and was not an efficient use of the available data. The same was true for censoring all patients prescribed orlistat at the end of 2010, when sibutramine use was suspended; HR 1.49 (95% CI 0.98–2.26).

Reducing the exposure period from 60 days following, the final medication dose to 15 days following the final dose produced a PS-adjusted HR of 1.87 (95% CI 1.23–12.84, *P*=0.03). The analysis for interaction between sibutramine and cardiovascular risk factors produced similar findings to the original analysis. There was weak evidence that those without cardiovascular disease had an elevated rate of MI/CVE if prescribed sibutramine (HR 1.62, 95% CI 0.98–2.67, *P*=0.058), whereas there was strong evidence that those with pre-existing disease were at increased risk (HR 4.96, (% CI 2.51–9.78, *P*=0.001).

### Rate of all-cause mortality

There were 152 deaths in the cohort during the exposure time (124.82 per 10^5^ PYAR, 95% CI 106.47–146.32). In the sibutramine exposed group, there were 10 deaths in 0.13 per 10^5^ PAYR compared with 142 in 1.09 per 10^5^ in the orlistat group. Crude HR was 0.50 (95% CI 0.26–0.99) This potential difference in mortality between sibutramine and orlistat patients was reduced following adjustment for age, sex, BMI, smoking, alcohol, coronary heart disease, CVD, peripheral vascular disease, T2D, other atheroma and hypertension, (HR 0.64, 95% CI 0.32–1.28). Similar results were seen with PS adjustment (Table 4). There was no clear evidence of increased rate of all-cause mortality in the group of patients taking sibutramine who had pre-existing cardiovascular or CVD (HR 1.82, 95% CI 0.53–6.21, *P*= 0.34). However, there was an increased rate of mortality in those with T2D and cardiac risk factors (HR 2.72, 95% CI 1.12–6.59–1.57, *P*=0.026). However, these analyses were underpowered and relate to death from a wide range of causes including many that sibutramine would not be expected to influence and should, therefore, be viewed as exploratory.

## Discussion

This large cohort study, which is representative of ‘real World' overweight and obese individuals registered with a GP in the United Kingdom,^22^ shows that sibutramine is associated with increased rate of MI/CVE. This increase is primarily driven by elevated rates in those with pre-existing cardiovascular disease (even though this group represented only 5% of the cohort), and those without pre-existing disease may have little or no increased risk. It also shows that sibutramine is unlikely to alter all-cause mortality overall, but there remains a possibility of an increased risk of mortality in a subgroup of people with diabetes and cardiovascular risk factors. Although limited by the non-random allocation of sibutramine or orlistat to patients, these findings are similar to SCOUT.^3^

Our group has previously shown the amount of weight loss associated with sibutramine use is marginal at best^9^ and in the context of a poor expected benefit, substantial potential harms are not tolerable. Our results confirm that the risk benefit balance in people with existing cardiovascular disease was negative but our findings in those without cardiovascular disease are less clear. We found no strong evidence of an increased risk of MI/CVE in this group but were unable to rule out a potential doubling of risk due to small patient numbers. Nonetheless, even if a causal effect exists in people without CVD, the absolute risk would remain low in this group.

### Strengths and limitations

The use of contemporaneous, representative medical records avoided the risk of potential biases relating to selection. Information bias should partially have been avoided by the use of prescribing data as exposure, in the UK GPs are responsible for all ongoing prescribing, which is detailed and well recorded in CPRD.^13^

The similarities between orlsitat and sibutramine treatment groups in terms of PS suggested that confounding by indication was unlikely to be a major issue in this study. Two important differences could be history of CVD and prescribing of statins. Indeed, people in the sibutramine group were less likely to have pre-existing CVD, and less likely to receive statins than those receiving orlistat. This is in keeping with the prescribing guidance for these drugs. Although we adjusted for this in our analyses, it is possible that residual confounding remained. However, this would tend to lead to an underestimate of the increased risk among sibutramine users. It is also possible that our PS model could have been improved by inclusion of extra baseline characteristics such as blood pressure, heart rate and lipid profile but this analysis was not possible, and we instead adjusted for correlates of these characteristics such as diagnosis of hypertension, prescribing of statins and a range of other cardiovascular health factors.

The study design reveals a temporal relationship between sibutramine exposure and MI/CVE, this relationship is strong in the pre-existing cardiovascular disease group, and a plausible and coherent causal mechanism for this effect has been identified.^8^

Exposure was defined as starting at the time of prescription issue and ending 60 days after the calculated end date of the final prescription. Poor adherence to prescribed drug regimens is a problem with all medication, and this is particularly true if side effects are unpleasant, as can be the case with sibutramine and orlistat.^23^ It is, therefore, likely that exposure time has been overestimated, which could lead to a result biased towards no effect. However, in the sibutramine exposed group the median period of usage was 0.38 years, suggesting that patients did re-present to their GP to collect further prescriptions. Also, the sensitivity analysis reducing the at risk period after the final medication date to 15 days found a similar increased rate in those exposed to sibutramine.

Although hard outcomes such as MI, CVE or death should be well recorded by GPs there is some evidence that CPRD underestimates the rate of these events by as much as 25%.^24^ Despite this there is no reason to assume events are differentially recorded for those individuals exposed to sibutramine, and so this may have resulted in reduced power, rather than incorrect estimates of effect. As well as this there may be errors in identifying acute events. Often a patient would have multiple codes representing acute MI or CVE on multiple dates in their record. When this occurred their full note history was searched for a suggestion that they had experienced a previous event, if so they were excluded. If there was no evidence of a previous event, the MI or CVE was recorded as occurring on the date of first entry of that code. However, this may be inaccurate if the patients had an event before the start of study follow-up, had recently moved practices or their event was coded late (from a hospital letter for instance).

### Conclusions and recommendations

Sibutramine, as used in routine clinical practice, was associated with an increased risk of acute cardiovascular and CVEs in patients with underlying cardiovascular disease. The absolute risk appears to be high in this group, confirming that the contraindications defined in sibutramine's licensing were important and appropriate.^1^ However, the lack of a clear increased risk in people without underlying cardiovascular disease and the overall low absolute rate of events in this group suggest it may have remained a suitable treatment option for patients with no history of cardiovascular disease.

## Figures and Tables

**Table 1:** Baseline characteristics by prescribing of sibutramine or orlistat

	*Sibutramine*	*Orlistat*	P*-value*^a^
Total, *N* (%)	23 927 (23.70)	77 047 (76.30)	
Follow-up time, years—median (IQR)	0.39 (0.24–0.63)	0.47 (0.16–1.68)	<0.001
Age at drug start, years—median (IQR)	43.44 (35.10–53.69)	47.00 (36.97–57.46)	<0.001
BMI at drug start, kg m^−2^—median (IQR)	36.52 (32.87–41.26)	36.33 (32.82–40.82)	0.001
	N *(%)*	N *(%)*	P-*value*^b^
Women	19709 (82.38)	58 458 (75.87)	<0.001
Cardiovascular disease history	759 (3.17)	4631 (6.01)	<0.001
Cerebrovascular disease history	304 (1.27)	1869 (2.43)	<0.001
Preexisting peripheral vascular disease	182 (0.76)	1025 (1.33)	<0.001
Other atheroma	4 (0.02)	39 (0.05)	0.026
Preexisting Type 2 diabetes mellitus	4130 (17.26)	14 667 (19.03)	<0.001
History of hypertension	5337 (22.30)	24 543 (31.85)	<0.001
			
*Prescribed*			
Oral anti-diabetic medication	2922 (12.21)	10 083 (13.08)	<0.001
Insulin	894 (3.74)	3131 (4.06)	0.024
Statin	3870 (16.17)	17 757 (23.04)	<0.001
			
*Smoking*			
Never smoked	9997 (41.77)	32 213 (41.80)	0.90
Ex-smoker	8891 (37.25)	28 704 (37.15)	
Current smoker	5046 (21.08)	16 142 (20.95)	
			
*Alcohol consumption*			
Non-drinker	4066 (16.99)	13 237 (17.18)	<0.001
Ex-drinker	1328 (5.55)	5079 (6.59)	
Rare (<2 units per day)	5891(24.61)	19 101 (24.79)	
Moderate (3–6 units per day)	10 946 (45.73)	32 906 (42.70)	
Heavy (>6 units per day)	1703 (7.12)	6736 (7.40)	

Abbreviation: IQR, interquartile range.

a From quantile regression.

b From *χ*^2^-test.

**Table 2:** Hazard ratios for incident MI/CVE from Cox regression

	N *patients*	*10*^*5*^ *PYAR*	N *events*	*Hazard ratio (95% CI)*	P-*value*^a^	*Heterogeneity*
*Primary analysis*						
Multivariable adjusted^b^						
Orlistat	77 047	1.092	220	1	0.018	
Sibutramine	23 927	0.126	34	1.65 (1.08–2.51)		
PS^c^ adjusted						
Orlistat	77 047	1.092	220	1	0.013	
Sibutramine	23 927	0.126	34	1.69 (1.12–2.56)		

*Stratified analyses*						
Cardiovascular disease						
Orlistat	6638	0.104	90	1	<0.00	*P*=0.0076
Sibutramine	1123	0.006	12	4.37 (2.21–8.64)	01	
No cardiovascular disease						
Orlistat	70 409	0.988	130	1	0.103	
Sibutramine	22 804	0.120	22	1.52 (0.92–2.48)		
Type 2 diabetes +1 cardiovascular risk factor						
Orlistat	12 346	0.176	95	1	0.005	*P*=0.53
Sibutramine	3109	0.018	9	2.81 (1.37–5.77)		
No type 2 diabetes						
Orlistat	64 701	0.916	125	1	0.002	
Sibutramine	20 818	0.108	25	2.18 (1.35–3.53)		

a From Wald test.

b Adjusted for age; sex; BMI; smoking status; alcohol use; history of: cardiovascular disease, cerebrovascular disease, peripheral vascular disease, other atheroma, type 2 diabetes and hypertension.

c Propensity score.

**Table 3:** Number needed to harm with sibutramine by cardiovascular disease risk group

	*NNH^a^ (95% CI) at 4 months*	*NNH*^a^ *(95% CI) at 12 months*
Cardiovascular disease	129 (57–360)	28 (12–77)
No cardiovascular disease	4809 (1690 to protective effect)	1749 (615 to protective effect)

a Number needed to harm, from estimated survivor function following Cox regression with PS adjustment.

**Table 4:** Hazard ratios for all-cause mortality from Cox regression

	N *patients*	*10*^*5*^ *PYAR*	N *events*	*Hazard ratio (95% CI)*	P*-value*^a^	*Heterogeneity*
*Primary analysis*						
* *Multivariable adjusted^b^						
Orlistat	77 047	1.092	142	1	0.212	
Sibutramine	23 927	0.126	10	0.64 (0.32–1.28)		
PS^c^ adjusted						
Orlistat	77 047	1.092	142	1	0.243	
Sibutramine	23 927	0.126	10	0.67 (0.34–1.32)		
						
*Stratified analyses*						
Cardiovascular disease						
Orlistat	6638	0.104	46	1	0.338	*P*=0.11
Sibutramine	1123	0.006	3	1.82 (0.26–1.29)		
						
No cardiovascular disease						
Orlistat	70 409	0.988	96	1	0.184	
Sibutramine	22 804	0.120	7	0.58 (0.26–1.29)		
						
Type 2 diabetes +1 cardiovascular risk factor						
Orlistat	12 346	0.176	65	1	0.026	*P*=0.0074
Sibutramine	3109	0.018	6	2.72 (1.12–6.59)		
						
No type 2 diabetes						
Orlistat	64 701	0.916	77	1	0.147	
Sibutramine	20 818	0.108	4	0.47 (0.17–1.31)		

a From Wald test.

b Adjusted for age; sex; BMI; smoking status; alcohol use; history of: cardiovascular disease, cerebrovascular disease, peripheral vascular disease, other atheroma, type 2 diabetes and hypertension.

c Propensity score.

## Appendix 1

Propensity score by drug exposure.
